# Association Between Pre-Existing Anti-Diphtheria Toxoid IgG and Immune Responses to a CRM_197_-Conjugated 14-Valent Pneumococcal Conjugate Vaccine in Infants: A Post Hoc Analysis of a Multicenter Phase III Trial

**DOI:** 10.3390/vaccines14100854

**Published:** 2026-09-28

**Authors:** Subhash Thuluva, Subbareddy Gunneri, Siddalingaiah Ningaiah, Vijay Yerroju, Rammohan Reddy Mogulla, Kamal Thammireddy, Shivani Desai, Venkatalakshmi Gundu, Atul Jindal, Pradeep Nanjappa, Bheemisetty S. Chakravarthy, Niranjana S. Mahantashetti, Savita Verma, Manish Narang, Jog Pramod Prabhakar

**Affiliations:** 1 Biological E. Limited, Hyderabad 500033, India; 2 All India Institute of Medical Sciences, Raipur 492099, India; 3 Cheluvamba Hospital, Mysore Medical College & Research Institute, Mysore 570001, India; 4 King George Hospital, Visakhapatnam 530002, India; 5 Karnataka Lingayat Education Society, Dr. Prabhakar Kore Hospital & Medical Research Centre, Belagavi 590010, India; 6 Pandit Bhagwat Dayal Sharma Post Graduate Institute of Medical Sciences, Rohtak 124001, India; 7 Guru Teg Bahadur Hospital, Delhi 110095, India; 8 Medipoint Hospital, Pune 411007, India

**Keywords:** pneumococcal conjugate vaccine, CRM_197_, maternal antibodies, pre-existing anti-diphtheria toxoid IgG, infant immunization, BE-PCV14, post hoc analysis

## Abstract

**Background/Objectives:** Maternally transferred antibodies protect infants early in life, but high concentrations can reduce immune responses to primary vaccination. CRM_197_ is a nontoxic mutant of diphtheria toxin that retains antigenic similarity to the native protein. We therefore investigated whether anti-diphtheria antibodies present before vaccination were related to responses against pneumococcal polysaccharides conjugated to CRM_197_. **Methods:** This post hoc analysis used data from a multicenter, randomized, single-blind phase III trial in which Indian infants received BE-PCV14/PNEUBEVAX 14^®^ at 6–8, 10–12, and 14–16 weeks of age (commonly referred to as 6–10–14 weeks). Baseline anti-diphtheria toxoid IgG concentrations measured at 6–8 weeks were assessed in relation to serotype-specific pneumococcal IgG responses 28 days after dose 3. We evaluated these associations using categorical and continuous analyses with false-discovery-rate correction for serotype-specific comparisons. **Results:** Baseline and post-primary results were available for 603 of 650 infants in the co-administration cohort. Post-primary pneumococcal IgG concentrations did not differ significantly across the baseline anti-diphtheria IgG categories. Individual fold rises differed nominally among baseline anti-diphtheria IgG categories for serotype 5, but the difference did not remain significant after multiplicity correction. In the adjusted continuous models, geometric mean ratios (GMRs) associated with each 2-fold increase in baseline anti-diphtheria IgG ranged from 0.978 to 1.011, with no statistically significant associations after false-discovery-rate correction. Analyses stratified by baseline pneumococcal IgG similarly showed no consistent evidence of reduced responses. **Conclusions:** In this cohort, pre-existing anti-diphtheria toxoid IgG measured before vaccination, which was likely maternally derived, was not associated with a consistent reduction in post-primary serotype-specific pneumococcal IgG responses to CRM_197_-conjugated BE-PCV14.

## 1. Introduction

Pneumococcal disease continues to cause substantial illness and death worldwide, particularly among children younger than 5 years in low- and middle-income countries [1]. The introduction of pneumococcal conjugate vaccines (PCVs) into childhood immunization programs has markedly reduced this burden [1]. The World Health Organization (WHO) recommends starting PCV vaccination at 6 weeks of age, using either a 3 + 0 or a 2 + 1 schedule [1,2]. India has incorporated PCV into its Universal Immunization Programme, reflecting the vaccine’s public-health importance [2].

In PCVs, capsular polysaccharides are covalently attached to a carrier protein so that infants and young children can mount a T-cell-dependent response. One commonly used carrier protein is CRM_197_, a nontoxic mutant of diphtheria toxin that is included in several licensed conjugate vaccines, including higher-valency PCVs [3,4]. Carrier-specific T-cell help contributes to the response to a conjugate vaccine. It is therefore possible that immunity already present against the carrier could alter the response to its linked polysaccharide antigen [5].

Antibodies transferred across the placenta provide important protection before an infant completes the primary vaccination series. At high concentrations, however, maternal antibodies may blunt the infant’s response to vaccination [6,7]. An individual-participant meta-analysis that included 7630 infants from 32 studies in 17 countries found an inverse relationship between maternal antibody concentrations and post-primary responses for 20 of 21 vaccine antigens. The same analysis found that responses to most antigens improved when the first vaccination was given at an older age [7]. At 6–8 weeks, most circulating anti-diphtheria antibody is expected to be maternal in origin, especially in settings where diphtheria-containing vaccines are routinely given during pregnancy [6,7,8].

Such interference with pneumococcal conjugate vaccination is biologically plausible, although published findings differ among studies and serotypes. Infants whose mothers received tetanus, diphtheria, and acellular pertussis (Tdap) vaccine during pregnancy have sometimes had lower post-primary geometric mean concentrations against selected PCV13 serotypes. Nevertheless, most infants reached accepted antibody thresholds, and differences generally became smaller after the booster dose [9,10].

Direct evidence relating infant anti-diphtheria antibody concentrations to responses to CRM_197_-conjugated PCVs remains limited, particularly in low- and middle-income settings and with schedules beginning at 6 weeks of age. The issue is pertinent to India, where a tetanus- and reduced-dose diphtheria-containing vaccine is routinely recommended in pregnancy, while infant PCV vaccination starts at 6 weeks of age [2,8]. In its 2024 post-validation assessment of maternal and neonatal tetanus elimination, WHO reported national Td2+ coverage of 83% and protection at birth of 92%, based on NFHS-5 data collected in 2019–2021 [11]. A substantial proportion of anti-diphtheria antibody in young Indian infants is therefore likely to have been acquired maternally.

BE-PCV14 (PNEUBEVAX 14^®^), developed by Biological E. Limited, is a CRM_197_-conjugated 14-valent PCV. In addition to the 12 serotypes shared with PCV13, it contains serotypes 22F and 33F. Infant studies have shown non-inferior immunogenicity to PCV13 and a comparable safety profile [12,13,14]. The vaccine is WHO-prequalified and is currently used in India’s Universal Immunization Programme for routine immunization of infants beginning at 6 weeks of age [2,15,16].

Using data from a multicenter phase III trial, we examined whether pre-existing anti-diphtheria toxoid IgG levels measured in infants at 6–8 weeks of age, before administration of a DTP-containing vaccine, were associated with serotype-specific IgG responses following the three-dose BE-PCV14 primary series. Our objective was to determine whether higher baseline anti-diphtheria toxoid IgG concentrations were associated with consistently lower pneumococcal antibody responses following the three-dose primary series.

## 2. Materials and Methods

### 2.1. Study Design and Participants

We performed a post hoc analysis of a prospective, randomized, active-controlled, single-blind phase III trial conducted at 29 Indian sites from August 2025 to February 2026. Study sites and their ethics committees are listed in Supplementary Materials File S1. The parent study compared Biological E. inactivated poliovirus vaccine (BE-IPV) with a licensed IPV comparator, given at 6, 10, and 14 weeks, and also assessed interference with vaccines administered at the same visits. Both IPV groups received the same DTwP-HepB-Hib, BE-PCV14, and oral rotavirus vaccine brands on an identical schedule. Since exposure to BE-PCV14 did not differ by IPV assignment, the two groups were combined for this analysis. This post hoc analysis was reported in accordance with the Strengthening the Reporting of Observational Studies in Epidemiology (STROBE) guidelines for cohort studies.

Healthy male and female infants aged 6–8 weeks were eligible if they had received only the recommended birth doses of bivalent oral poliovirus vaccine, Bacille Calmette-Guérin (BCG) vaccine, and hepatitis B vaccine, and if a parent or legally acceptable representative provided written informed consent. Key exclusion criteria were previous receipt of a DTP-containing vaccine, IPV, or PCV; confirmed or suspected poliomyelitis, diphtheria, tetanus, pertussis, or pneumococcal disease; known immunosuppression; previous receipt of intravenous immunoglobulin; and known or suspected allergy to any study vaccine. A total of 650 infants were enrolled in the co-administered vaccine immunogenicity cohort. Full eligibility criteria are provided in Supplementary Materials File S2.

### 2.2. Ethics and Registration

The protocol of the original trial was approved by the institutional ethics committee at each participating site and by the Central Drugs Standard Control Organization, India’s national regulatory authority. The trial was prospectively registered with the Clinical Trials Registry—India (CTRI/2025/05/086953; registered on 14 May 2025). Written informed consent was obtained from a parent or legally acceptable representative before any study procedure. The trial was conducted in accordance with the Declaration of Helsinki, the International Council for Harmonisation Good Clinical Practice, and applicable regulatory requirements.

Neither patients nor members of the public participated in the design, conduct, or reporting of the study. There were no major protocol deviations. Visits for a small number of participants fell outside the protocol-specified window, but investigators judged that these deviations had no clinically important effect on participant safety or study validity; they were reported to the relevant ethics committees. The approved protocol was not amended.

### 2.3. Randomization and Masking

Eligible infants were randomized 1:1 to receive BE-IPV or the licensed comparator IPV through a validated interactive web response system. An independent statistician from the external biostatistics service provider generated the randomization schedule using PROC PLAN in SAS version 9.4 and had no role in participant enrollment, clinical assessment, or laboratory testing. Investigators enrolled participants and assigned treatment through the interactive web response system without access to the underlying sequence. All participants received co-administered BE-PCV14, DTwP-HepB-Hib vaccine, and oral rotavirus vaccine.

The parent trial was single-blind: parents or legally acceptable representatives were unaware of IPV assignment, whereas investigators were unblinded because the study vaccines retained their commercial labels. Vaccine preparation and syringe loading were performed outside the participant’s and parent’s view. Laboratory personnel remained blinded to treatment allocation and tested coded serum samples.

### 2.4. Procedures

The study included four scheduled visits. At Day 0 (Visit 1), infants were screened and enrolled, baseline blood was collected, and the first dose of BE-IPV or comparator IPV was administered with BE-PCV14, DTwP-HepB-Hib vaccine, and oral rotavirus vaccine. The second and third doses were administered on Days 28 and 56, respectively. Post-primary blood was collected on Day 84, 28 days after dose 3. A window of +7 days was permitted for Visits 2–4.

Approximately 5.0 mL of blood was collected at Visits 1 and 4. Serum was separated and stored at −20 °C to −80 °C until analysis.

The present analysis included baseline anti-diphtheria toxoid IgG and baseline and post-primary serotype-specific anti-pneumococcal capsular polysaccharide IgG concentrations. Anti-diphtheria toxoid IgG was measured before dose 1 using a quantitative IgG enzyme-linked immunosorbent assay (EUROIMMUN, Lübeck, Germany) at Dr. Dangs Lab LLP, New Delhi, India. The lower limit of quantification for the assay was 0.01 IU/mL.

Serotype-specific anti-pneumococcal capsular polysaccharide IgG concentrations for the 14 vaccine serotypes were measured at Visits 1 and 4 at the Central Research Laboratory, Kempegowda Institute of Medical Sciences, Bengaluru, using a standardized, validated enzyme-linked immunosorbent assay based on the WHO reference method. The 1st International Standard for Human Anti-pneumococcal Capsular Reference Serum, 007sp, supplied by the National Institute for Biological Standards and Control, United Kingdom, served as the reference standard. Dilution-corrected antibody concentrations were estimated using a 4-parameter logistic model. Detailed assay procedures, including the serotype-specific pre-absorption conditions, are provided in Supplementary Materials File S6. The same assay platform was used in earlier BE-PCV14 studies that supported licensure and WHO prequalification.

All assays were performed in certified and/or Good Clinical Laboratory Practice-compliant laboratories. Other immunogenicity assessments from the parent trial are not reported here.

### 2.5. Vaccines and Immunization Procedures

Each 0.5 mL dose of PNEUBEVAX 14^®^ (BE-PCV14) contained 3 µg of serotype 1 polysaccharide; 2.2 µg of each of serotypes 3, 4, 5, 7F, 9V, 14, 18C, 19A, 19F, 22F, 23F, and 33F; and 4.4 µg of serotype 6B polysaccharide. The polysaccharides were conjugated to 20–50 µg of CRM_197_ and adsorbed onto ≤0.75 mg of aluminum phosphate; each dose also contained 4 mg of 2-phenoxyethanol.

The compositions of the co-administered DTwP-HepB-Hib, IPV, and oral rotavirus vaccines are provided in Supplementary Materials S3.

BE-PCV14 (0.5 mL) and DTwP-HepB-Hib vaccine (0.5 mL) were administered intramuscularly at separate sites in the anterolateral thigh, at least 1 inch apart, at 6, 10, and 14 weeks of age. BE-IPV or comparator IPV (0.5 mL) was administered intramuscularly in the contralateral thigh. Oral rotavirus vaccine (0.5 mL) was administered orally at the same visits.

### 2.6. Outcomes

Outcomes for this analysis were baseline anti-diphtheria toxoid IgG concentration; the distribution of infants across predefined baseline categories (<0.1, 0.1 to <1.0, and ≥1.0 IU/mL); serotype-specific anti-pneumococcal capsular polysaccharide IgG concentration 28 days after dose 3; geometric mean fold rise (GMFR) from baseline to Day 84; and the adjusted geometric mean ratio (GMR) for the post-primary pneumococcal IgG response associated with each 2-fold increase in baseline anti-diphtheria toxoid IgG.

### 2.7. Statistical Analysis

A separate statistical analysis plan for this post hoc analysis was finalized before the analyses were performed. Baseline anti-diphtheria toxoid IgG measured before the first vaccine dose was the exposure of interest. Its relationship with post-primary pneumococcal IgG was examined using predefined concentration categories and as a continuous variable in regression analyses. The continuous regression analysis was the principal inferential analysis. Tertile-based analyses and analyses stratified by baseline pneumococcal IgG were performed as sensitivity analyses.

For the categorical analysis, baseline anti-diphtheria toxoid IgG concentrations were classified as low (<0.1 IU/mL), medium (0.1 to <1.0 IU/mL), or high (≥1.0 IU/mL), based on established serological thresholds for diphtheria antitoxin and concentration categories reported previously [17,18].

GMCs and GMFRs were calculated using log-transformed values, and two-sided 95% confidence intervals were calculated on the log scale and back-transformed. Anti-diphtheria toxoid IgG concentrations below the lower limit of quantification were assigned a value of 0.005 IU/mL, corresponding to one-half of the lower limit of quantification, for statistical analyses. Post-primary serotype-specific IgG concentrations and individual fold rises were compared across the predefined baseline anti-diphtheria IgG categories using the Kruskal–Wallis test. These comparisons were unadjusted. As a sensitivity analysis, baseline anti-diphtheria toxoid IgG concentrations were categorized into tertiles of the observed distribution, and the same analytical approach was applied.

As an additional descriptive analysis, the number and percentage of infants with post-primary serotype-specific pneumococcal IgG concentrations below the seroresponse threshold of 0.35 µg/mL were summarized according to the predefined baseline anti-diphtheria IgG groups. The unadjusted association between baseline anti-diphtheria toxoid IgG and post-primary serotype-specific pneumococcal IgG concentrations was assessed using Spearman rank correlation. Separate correlations were calculated for each of the 14 pneumococcal serotypes. Individual participant data were also displayed as serotype-specific scatterplots, with both antibody concentrations shown on logarithmic scales. Pairwise correlations among post-primary IgG responses to the 14 pneumococcal serotypes were assessed using Spearman rank correlation. For each infant, we also calculated the number of serotypes with post-primary IgG < 0.35 µg/mL. The number of serotypes below this threshold was compared across the predefined baseline anti-diphtheria IgG groups using the Kruskal–Wallis test and correlated with baseline anti-diphtheria IgG using Spearman rank correlation.

Separate linear regression models were fitted for each pneumococcal serotype, following an approach similar to that described by Voysey et al. [7]. Post-primary serotype-specific pneumococcal IgG, baseline anti-diphtheria toxoid IgG, and baseline serotype-specific pneumococcal IgG concentrations were log2-transformed before analysis. For each serotype, post-primary pneumococcal IgG was the dependent variable, baseline anti-diphtheria toxoid IgG was the explanatory variable of interest, and baseline serotype-specific pneumococcal IgG and the IPV treatment group were included as covariates. The regression coefficient for baseline anti-diphtheria toxoid IgG and its 95% confidence interval were exponentiated using base 2 (2^β) and expressed as the adjusted geometric mean ratio (GMR) of post-primary pneumococcal IgG associated with each 2-fold increase in baseline anti-diphtheria toxoid IgG.

A stratified sensitivity analysis was performed according to baseline serotype-specific pneumococcal IgG concentration (<0.35 or ≥0.35 µg/mL). The 0.35 µg/mL threshold was selected because it is the WHO reference IgG concentration commonly used in the evaluation of pneumococcal conjugate vaccine immunogenicity [19]. In the present analysis, this threshold was used only to stratify infants according to baseline pneumococcal antibody concentration and was not intended to define individual protection. Within each stratum, post-primary pneumococcal IgG and baseline anti-diphtheria toxoid IgG concentrations were log2-transformed, and the regression models were adjusted for the IPV treatment group.

All *p* values were two-sided. The Benjamini–Hochberg procedure was used to control the false discovery rate across the 14 serotype-specific comparisons within each analysis. For the categorical and tertile-based analyses, multiplicity adjustment was applied separately to post-primary IgG concentrations and individual fold rises. For the stratified sensitivity analysis, false-discovery-rate adjustment was performed across the 28 stratum-specific serotype analyses. The Spearman correlation analysis was descriptive, and no multiplicity adjustment was applied to these correlations.

No imputation was performed for missing values. Analyses were restricted to infants with baseline anti-diphtheria toxoid IgG and evaluable serotype-specific pneumococcal IgG measurements at both baseline and Day 84.

All statistical analyses were performed using SAS version 9.4 (SAS Institute Inc., Cary, NC, USA).

### 2.8. Sample Size

A separate sample size was not calculated for this post hoc analysis. Instead, the sample was defined by the number of participants available from the parent phase III trial. Infants were included if the baseline anti-diphtheria toxoid IgG and evaluable serotype-specific pneumococcal IgG results were available both at baseline and 28 days after dose 3. These requirements were met by 603 of the 650 enrolled infants.

As this was an exploratory analysis, it was not prospectively powered to test associations for individual pneumococcal serotypes. The three predefined baseline anti-diphtheria IgG categories included 191, 307, and 105 infants, respectively. For interpretation of the continuous analyses, a GMR of 0.80 would correspond to a 20% lower post-primary pneumococcal IgG concentration for each 2-fold increase in baseline anti-diphtheria IgG. This value was used only as a descriptive reference and was not an established clinical or non-inferiority margin.

### 2.9. Data Management and Independent Statistical Analysis

Clinical data from the parent trial were managed and validated by an external vendor in accordance with the trial data management plan. For this post hoc analysis, an independent statistician who was not employed by the sponsor analyzed the finalized analysis dataset according to a separate statistical analysis plan. The sponsor and study team participated in the clinical interpretation of the findings, while data management and statistical analyses were conducted independently.

## 3. Results

### 3.1. Demographics and Baseline Characteristics

Participant disposition is shown in Figure 1. The co-administration cohort comprised 650 healthy Indian infants aged 6–8 weeks, randomized equally to BE-IPV or comparator IPV. Every infant was scheduled to receive the BE-PCV14, DTwP-HepB-Hib vaccine, and oral rotavirus vaccine at 6, 10, and 14 weeks. Ten infants assigned to BE-IPV and 12 assigned to comparator IPV discontinued before completing the study. Twenty-five others could not be included in this post hoc analysis because of insufficient serum samples for pneumococcal immunogenicity testing. The final analysis population therefore consisted of 603 infants with evaluable baseline anti-diphtheria toxoid IgG and post-primary pneumococcal IgG results.

Baseline demographic and anthropometric characteristics are summarized in Table 1. In the 650-infant co-administration cohort, mean (SD) age at enrollment was 47.8 (4.20) days, and 318 infants (48.92%) were female. Mean (SD) weight, height, and body mass index were 4312.5 (610.53) g, 53.7 (2.23) cm, and 15.0 (2.12) kg/m^2^, respectively. All participants were Asian and of Indian nationality.

### 3.2. Baseline Anti-Diphtheria Toxoid IgG Distribution

Among the 603 evaluable infants, 191 had baseline anti-diphtheria toxoid IgG concentrations < 0.1 IU/mL, 307 had concentrations from 0.1 to <1.0 IU/mL, and 105 had concentrations ≥ 1.0 IU/mL. Corresponding baseline geometric mean concentrations were 0.03, 0.39, and 2.36 IU/mL.

### 3.3. Post-Primary Pneumococcal IgG Responses by Predefined Baseline Anti-Diphtheria IgG Categories

None of the 14 serotypes showed a significant difference in post-primary pneumococcal IgG concentration among the three baseline anti-diphtheria toxoid IgG categories (all *p* > 0.05). Unadjusted *p* values ranged from 0.180 for serotype 7F to 0.979 for serotype 19A. GMFRs were generally similar as well. For serotype 5, individual fold rises differed nominally among the three categories (Kruskal–Wallis *p* = 0.007), but this finding was no longer significant after false-discovery-rate correction (q = 0.105). Detailed serotype-specific results are presented in Table 2.

The proportion of infants achieving the seroresponse threshold of ≥0.35 µg/mL was similar across the baseline anti-diphtheria IgG groups. For 12 of the 14 serotypes, at least 97% of infants in each group achieved the threshold. For serotype 3, the corresponding proportions were 88.5%, 87.9%, and 85.7% in the low, medium, and high baseline anti-diphtheria IgG groups, respectively, and for serotype 4 they were 93.2%, 94.1%, and 93.3%. The proportion of responses below the seroresponse threshold was also similar across the three groups (Supplementary Table S7).

### 3.4. Continuous-Model Analysis

In the continuous analysis, log2-transformed post-primary pneumococcal IgG concentration was modeled as a function of log2-transformed baseline anti-diphtheria toxoid IgG, with adjustment for log2-transformed baseline serotype-specific pneumococcal IgG and IPV treatment group.

For every serotype, the adjusted GMR associated with a doubling of baseline anti-diphtheria toxoid IgG was near 1.0 (range, 0.978–1.011). The estimates and their 95% confidence intervals did not indicate a consistent reduction in response, and all associations were nonsignificant after false-discovery-rate correction. The lowest point estimate was observed for serotype 3 (GMR 0.978 per 2-fold increase), but the association was not statistically significant. Adjusted GMRs and 95% confidence intervals are presented in Table 3 and Figure 2.

### 3.5. Unadjusted Association Between Baseline Anti-Diphtheria IgG and Post-Primary Pneumococcal IgG

In the unadjusted analysis, there was no apparent monotonic association between baseline anti-diphtheria toxoid IgG and post-primary pneumococcal IgG concentrations. Spearman rank correlation coefficients were close to zero for all 14 serotypes, ranging from −0.049 to 0.049, and none of the correlations were statistically significant (Figure 3). The distribution of individual responses similarly showed no consistent tendency toward lower post-primary pneumococcal IgG concentrations with increasing baseline anti-diphtheria IgG. Pairwise correlations among post-primary IgG responses to the 14 pneumococcal serotypes were generally low (Spearman ρ, 0.024–0.271; median, 0.119; Supplementary Table S8). Most infants had no serotype with a post-primary IgG concentration < 0.35 µg/mL. The median number of serotypes below this threshold was 0 in all three baseline anti-diphtheria IgG groups, with mean values of 0.23, 0.25, and 0.27 in the low, medium, and high groups, respectively. There was no significant difference among the three groups (*p* = 0.710), and the number of serotypes below 0.35 µg/mL was not associated with baseline anti-diphtheria IgG (ρ = 0.054, *p* = 0.184).

### 3.6. Tertile-Based Sensitivity Analysis

Results were similar when baseline anti-diphtheria IgG was divided into tertiles. The tertile boundaries were ≤0.1219, >0.1219 to ≤0.6143, and >0.6143 IU/mL. Post-primary serotype-specific IgG concentrations and GMFRs were comparable across the three groups. Individual fold rises for serotype 5 differed nominally among tertiles (*p* = 0.033), but the difference was no longer significant after false-discovery-rate correction (q = 0.465). Across serotypes, GMFRs did not show a consistent decrease with increasing baseline anti-diphtheria IgG.

### 3.7. Stratified Sensitivity Analysis by Baseline Pneumococcal IgG Concentration

When the analysis was stratified by baseline serotype-specific pneumococcal IgG concentration (<0.35 or ≥0.35 µg/mL), baseline anti-diphtheria toxoid IgG was not significantly associated with post-primary pneumococcal IgG for any serotype. The stratum-specific GMRs were close to 1.0, and none of the associations were significant either before or after false-discovery-rate correction. Detailed results are shown in Supplementary Table S5.

## 4. Discussion

In this post hoc analysis, pre-existing anti-diphtheria toxoid IgG measured at baseline was not consistently associated with post-primary pneumococcal IgG responses in infants who received three doses of BE-PCV14. In the principal continuous analysis, adjusted GMRs associated with each 2-fold increase in baseline anti-diphtheria IgG were close to 1.0 for all 14 serotypes, with no statistically significant associations after correction for multiple comparisons. The same pattern was seen in the unadjusted analysis, with Spearman correlations close to zero across all 14 serotypes and no evident relationship in the individual participant data.

Similar findings were observed in the predefined categories and in the tertile-based and baseline pneumococcal IgG-stratified sensitivity analyses. For serotype 5, individual fold rises differed nominally in the predefined-category and tertile-based analyses, but these differences were no longer significant after correction for multiple comparisons. Overall, there was no consistent trend toward lower pneumococcal IgG responses with increasing baseline anti-diphtheria IgG.

Such an interaction is biologically plausible. Maternal antibodies can attenuate responses to several infant vaccine antigens [6,7]. PCVs also rely on carrier-protein-specific T-cell help to elicit T-cell-dependent immunity [4,5]. Circulating anti-diphtheria IgG may therefore not directly reflect the carrier-specific T-cell help involved in the response to CRM_197_-conjugated polysaccharides. This may partly explain why increasing baseline anti-diphtheria IgG was not associated with lower pneumococcal IgG responses in this study. However, because CRM_197_ is derived from diphtheria toxin, pre-existing immunity to the carrier could still influence responses to the conjugated polysaccharides, including through mechanisms such as carrier-induced epitopic suppression [5,20]. Carrier-specific cellular responses were not measured, so this possibility cannot be assessed directly from the present data.

Studies of maternal Tdap vaccination have reported lower post-primary responses to some PCV13 serotypes [9,10]. Maertens et al. observed lower geometric mean concentrations for several serotypes after the primary series, although most infants still reached accepted thresholds and the differences narrowed after boosting [9]. Abu-Raya et al. found a similar serotype-dependent pattern in an individual-participant data meta-analysis [10]. Data from a CRM_197_-conjugated PCV7 efficacy trial have also suggested that maternal pneumococcal antibody can affect early infant responses [21]. Those studies, however, focused on maternal vaccination or maternal and infant pneumococcal antibodies. In contrast, our analysis examined the infant’s anti-diphtheria toxoid IgG concentration rather than maternal vaccination status or maternal pneumococcal antibody.

The baseline anti-diphtheria IgG concentrations observed in this cohort were within the range reported in infants following maternal Tdap vaccination. Published studies have reported infant anti-diphtheria IgG concentrations of approximately 0.55 IU/mL at 7 weeks and 2.6 IU/mL at 2 months after maternal Tdap vaccination [22,23]. In our cohort, the baseline GMCs in the low, medium, and high groups were 0.03, 0.39, and 2.36 IU/mL, respectively.

Our analysis differs from that earlier work in several respects. We measured anti-diphtheria toxoid IgG in infants immediately before vaccination rather than using maternal vaccination status as a proxy. Vaccination started at 6 weeks and followed the 6–10–14-week schedule used in India and other low- and middle-income settings [1,2]. All infants received the same BE-PCV14 formulation, and serotype-specific antibody was measured using a standardized WHO reference assay.

These findings are relevant to settings where tetanus- and diphtheria-containing vaccines are administered during pregnancy and infant PCV vaccination begins early. Across the baseline antibody concentrations represented in this cohort, higher anti-diphtheria IgG was not accompanied by a general reduction in BE-PCV14 responses. The findings therefore do not suggest a need to change established maternal immunization practices because of concerns about potential interference with infant BE-PCV14 responses. However, this study was not designed to compare maternal immunization schedules and therefore cannot inform recommendations regarding the timing or choice of maternal vaccination.

The study benefits from a relatively large infant cohort, a common vaccination schedule, direct measurement of anti-diphtheria toxoid IgG before vaccination, and standardized serological testing. Adjustment for baseline serotype-specific pneumococcal IgG and agreement among several analytical approaches further strengthen the interpretation.

Several limitations should be considered. We did not collect maternal vaccination histories and therefore could not confirm that the infant anti-diphtheria antibody was maternally derived. Anti-diphtheria toxoid IgG was used as a marker of pre-existing carrier-related immunity potentially relevant to CRM_197_; antibodies specific to CRM_197_ were not measured directly. This post hoc analysis was not powered in advance to detect small effects for individual serotypes after adjustment for multiple comparisons. We measured binding IgG but not functional opsonophagocytic activity, and follow-up was limited to the post-primary time point. Consequently, the study does not address booster responses, persistence, or immune memory. Future studies could combine maternal vaccination records with paired maternal and cord samples, measures of placental transfer, and functional antibody assays.

## 5. Conclusions

In Indian infants who received the BE-PCV14 primary series at 6, 10, and 14 weeks, higher baseline anti-diphtheria toxoid IgG was not associated with a consistent reduction in post-primary serotype-specific pneumococcal IgG responses. This interpretation was unchanged in categorical, continuous, and stratified analyses. Within the range of baseline anti-diphtheria toxoid IgG concentrations observed in this cohort, there was no evidence that pre-existing anti-diphtheria antibody reduced the primary-series immunogenicity of the CRM_197_-conjugated BE-PCV14 vaccine.

## Figures and Tables

**Figure 1:**
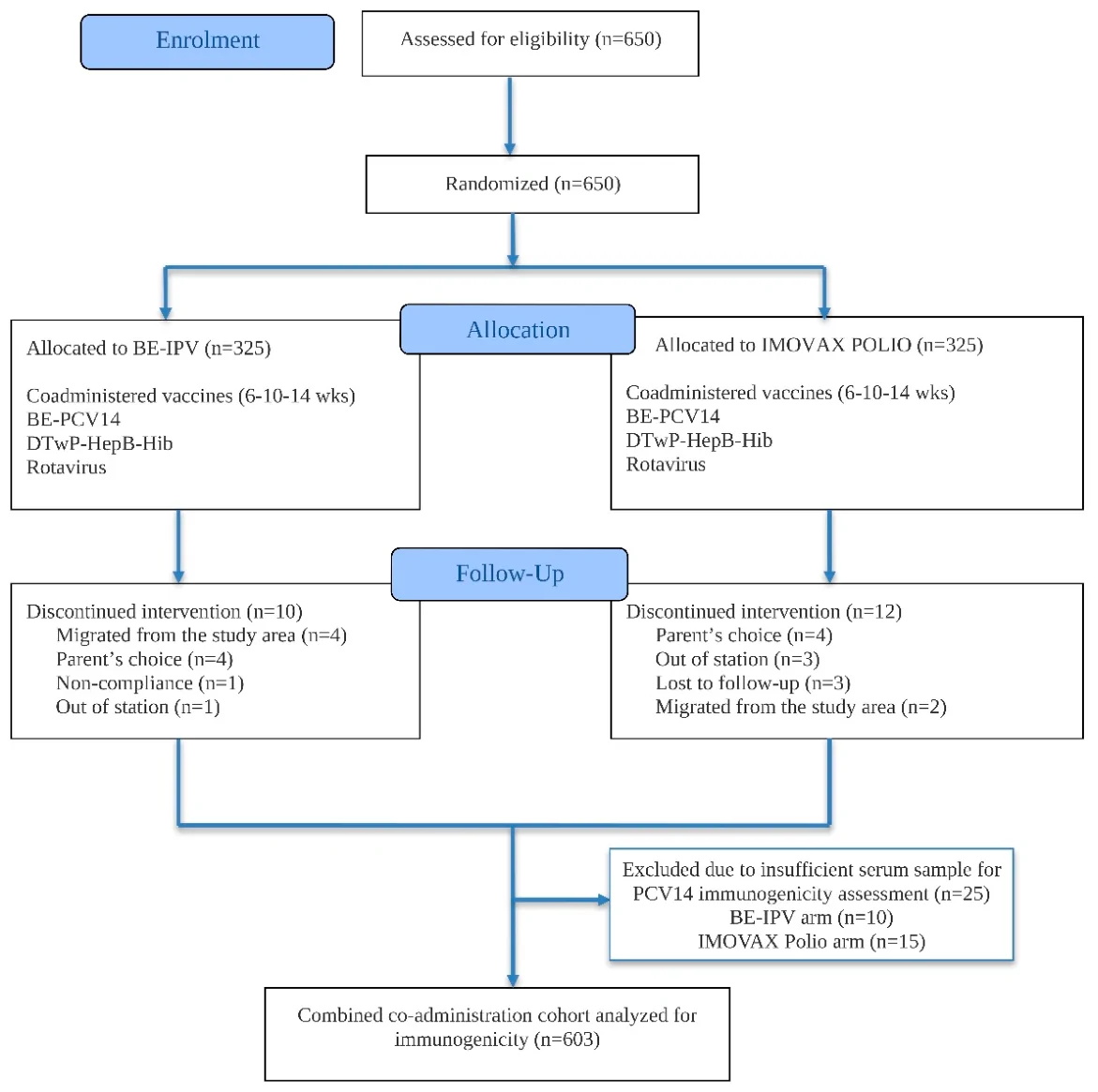
Participant disposition and analysis population. BE-IPV, Biological E. inactivated poliovirus vaccine; BE-PCV14, Biological E. 14-valent pneumococcal conjugate vaccine; DTwP-HepB-Hib, diphtheria, tetanus, whole-cell pertussis, hepatitis B, and *Haemophilus influenzae* type b vaccine; IMOVAX POLIO, comparator inactivated poliovirus vaccine; n, number of participants.

**Figure 2:**
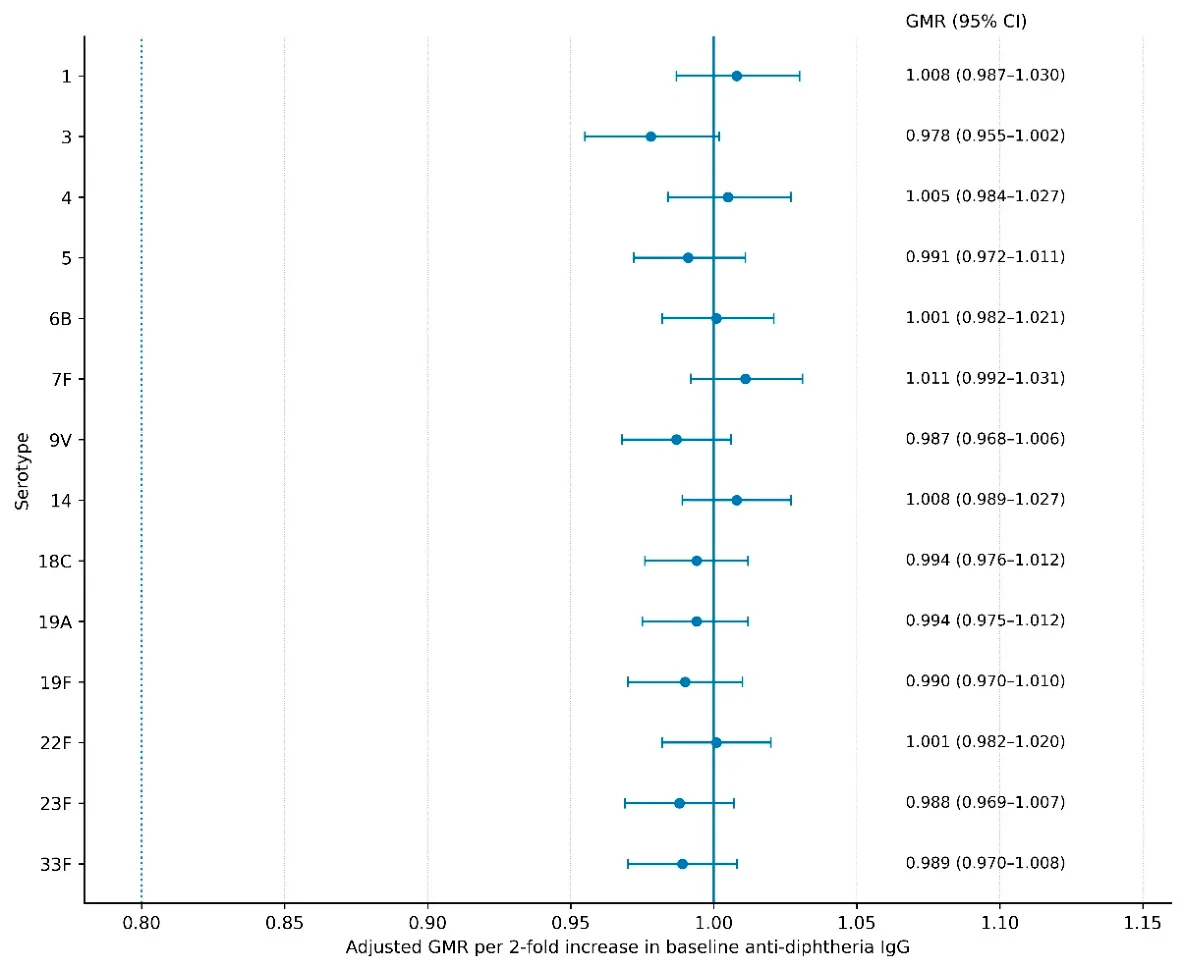
Association between baseline anti-diphtheria toxoid IgG and post-primary pneumococcal IgG responses. Points represent adjusted geometric mean ratios (GMRs), and horizontal bars represent 95% confidence intervals (CIs). The solid vertical line at 1.0 indicates no association; the dotted vertical line at 0.80 is shown as a descriptive reference corresponding to a 20% reduction in post-primary pneumococcal IgG concentration. GMR, geometric mean ratio; CI, confidence interval; IgG, immunoglobulin G.

**Figure 3:**
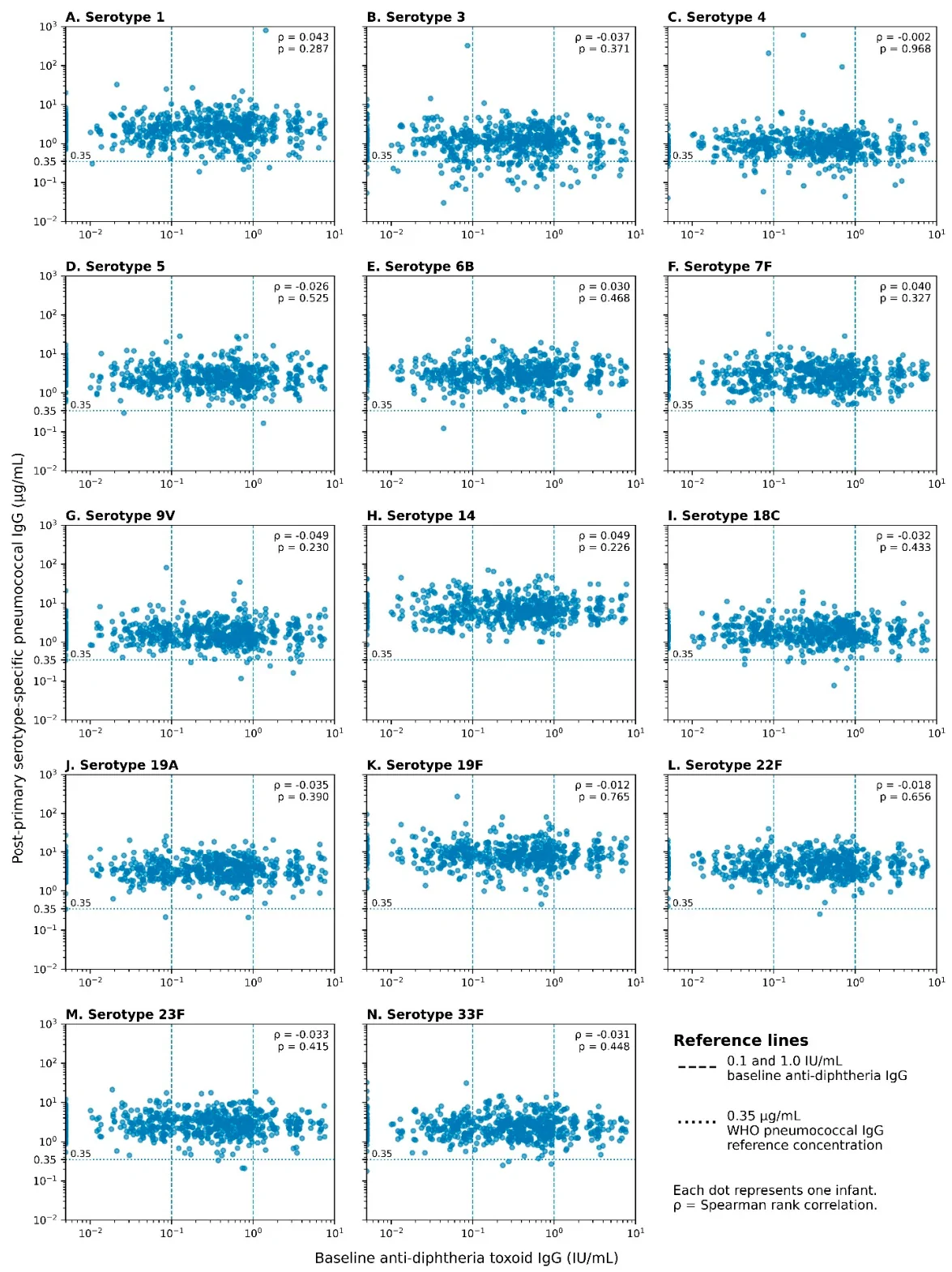
Association between baseline anti-diphtheria toxoid IgG and post-primary serotype-specific pneumococcal IgG concentrations. Each panel shows the relationship between baseline anti-diphtheria toxoid IgG and post-primary pneumococcal IgG concentration for one BE-PCV14 serotype: (**A**) serotype 1; (**B**) serotype 3; (**C**) serotype 4; (**D**) serotype 5; (**E**) serotype 6B; (**F**) serotype 7F; (**G**) serotype 9V; (**H**) serotype 14; (**I**) serotype 18C; (**J**) serotype 19A; (**K**) serotype 19F; (**L**) serotype 22F; (**M**) serotype 23F; and (**N**) serotype 33F. Each point represents an individual infant. Both antibody concentrations are shown on logarithmic scales. Vertical dashed lines indicate baseline anti-diphtheria toxoid IgG concentrations of 0.1 and 1.0 IU/mL, corresponding to the predefined concentration categories. The horizontal dotted line indicates the WHO reference pneumococcal IgG concentration of 0.35 µg/mL and is not intended to define individual protection. Spearman rank correlation coefficients (ρ) and corresponding *p* values are shown for each serotype.

**Table 1:** Demographics and baseline characteristics of study participants in the co-administration cohort.

Parameter	Combined Co-Administration Cohort
**Age, days**	
N	650
Mean (SD)	47.8 (4.20)
Min, Max	42; 56
**Sex, n (%)**	
Female	318 (48.92%)
Male	332 (51.08%)
**Weight, grams**	
N	650
Mean (SD)	4312.5 (610.53)
Min, Max	3300; 6900
**Height, cm**	
N	650
Mean (SD)	53.7 (2.23)
Min, Max	46; 61
**BMI, kg/m^2^**	
N	650
Mean (SD)	15.0 (2.12)
Min, Max	10; 24
**Nationality, n (%)**	
Indian	650 (100%)

N, total number of participants; n, number of participants in the specified category; SD, standard deviation; Min, minimum; Max, maximum; cm, centimeter; BMI, body mass index. BMI was calculated as weight (kg)/[length (m)]^2^. Bold text indicates parameter headings.

**Table 2:** Post-primary vaccination serotype-specific pneumococcal IgG GMCs (µg/mL) and GMFRs by predefined baseline anti-diphtheria IgG groups.

Serotype	Post-Vac GMC (95% CI) in Low Anti-D IgG (n = 191)	Post-Vac GMC (95% CI) in Medium Anti-D IgG (n = 307)	Post-Vac GMC (95% CI) in High Anti-D IgG (n = 105)	*p* Value (Post-IgG)	GMFR (95% CI), Low Anti-D IgG	GMFR (95% CI), Medium Anti-D IgG	GMFR (95% CI), High Anti-D IgG	*p* Value (Fold Rise)
1	2.23 (2.01, 2.46)	2.35 (2.15, 2.57)	2.45 (2.05, 2.92)	0.258	3.72 (3.32, 4.17)	3.98 (3.61, 4.39)	4.28 (3.59, 5.10)	0.399
3	1.09 (0.93, 1.27)	1.08 (0.98, 1.19)	0.98 (0.83, 1.17)	0.719	4.28 (3.73, 4.91)	4.17 (3.76, 4.63)	3.88 (3.24, 4.65)	0.713
4	0.87 (0.79, 0.97)	0.92 (0.84, 1.00)	0.86 (0.76, 0.98)	0.740	3.80 (3.40, 4.24)	3.99 (3.61, 4.40)	3.93 (3.28, 4.70)	0.765
5	2.55 (2.32, 2.80)	2.40 (2.22, 2.58)	2.56 (2.23, 2.94)	0.266	4.58 (4.07, 5.17)	3.79 (3.43, 4.20)	4.65 (3.82, 5.65)	0.007
6B	2.99 (2.72, 3.29)	3.03 (2.81, 3.26)	3.29 (2.85, 3.79)	0.330	3.81 (3.38, 4.29)	3.60 (3.28, 3.96)	4.26 (3.57, 5.09)	0.128
7F	2.47 (2.23, 2.73)	2.76 (2.55, 2.98)	2.55 (2.26, 2.89)	0.180	3.64 (3.26, 4.06)	4.02 (3.65, 4.44)	3.89 (3.31, 4.59)	0.607
9V	1.77 (1.60, 1.95)	1.64 (1.52, 1.76)	1.59 (1.38, 1.83)	0.572	4.11 (3.64, 4.64)	3.70 (3.36, 4.08)	4.10 (3.48, 4.81)	0.244
14	6.64 (5.96, 7.39)	6.88 (6.36, 7.43)	6.89 (6.14, 7.73)	0.596	3.78 (3.33, 4.29)	3.44 (3.14, 3.77)	3.69 (3.19, 4.28)	0.299
18C	1.88 (1.73, 2.05)	1.71 (1.59, 1.84)	1.73 (1.53, 1.95)	0.336	3.31 (2.96, 3.71)	3.48 (3.18, 3.80)	3.21 (2.74, 3.76)	0.620
19A	3.54 (3.22, 3.90)	3.49 (3.25, 3.74)	3.45 (3.04, 3.91)	0.979	3.72 (3.32, 4.17)	3.54 (3.24, 3.88)	3.85 (3.25, 4.57)	0.752
19F	8.93 (8.04, 9.91)	7.94 (7.31, 8.63)	8.49 (7.59, 9.50)	0.306	3.88 (3.38, 4.45)	3.62 (3.31, 3.96)	3.48 (3.01, 4.02)	0.976
22F	4.63 (4.22, 5.09)	4.54 (4.22, 4.90)	4.30 (3.82, 4.83)	0.677	3.85 (3.37, 4.41)	3.60 (3.27, 3.96)	3.90 (3.32, 4.59)	0.605
23F	2.94 (2.68, 3.23)	2.71 (2.51, 2.92)	2.81 (2.50, 3.17)	0.611	4.12 (3.62, 4.69)	3.50 (3.20, 3.82)	3.99 (3.33, 4.77)	0.168
33F	2.42 (2.19, 2.67)	2.33 (2.15, 2.52)	2.45 (2.17, 2.77)	0.650	3.91 (3.44, 4.43)	3.88 (3.53, 4.27)	4.12 (3.45, 4.91)	0.856

Vac, vaccination; anti-D, anti-diphtheria toxoid IgG; GMFR, geometric mean fold rise. Diphtheria antibody groups were defined according to baseline anti-diphtheria toxoid IgG concentrations. The baseline (pre-vaccination) anti-diphtheria GMC was 0.03 IU/mL in the low anti-D IgG group, 0.39 IU/mL in the medium anti-D IgG group, and 2.36 IU/mL in the high anti-D IgG group. *p* values were obtained using the Kruskal–Wallis test applied to post-primary IgG concentrations and individual fold rises, respectively.

**Table 3:** Continuous-model analysis of baseline anti-diphtheria IgG and post-primary pneumococcal IgG responses.

Serotype	GMR per 2-Fold Increase in Baseline Anti-Diphtheria IgG (95% CI)	*p* Value	FDR q
1	1.008 (0.987, 1.030)	0.451	0.667
3	0.978 (0.955, 1.002)	0.071	0.667
4	1.005 (0.984, 1.027)	0.638	0.744
5	0.991 (0.972, 1.011)	0.370	0.667
6B	1.001 (0.982, 1.021)	0.888	0.926
7F	1.011 (0.992, 1.031)	0.241	0.667
9V	0.987 (0.968, 1.006)	0.185	0.667
14	1.008 (0.989, 1.027)	0.430	0.667
18C	0.994 (0.976, 1.012)	0.524	0.667
19A	0.994 (0.975, 1.012)	0.488	0.667
19F	0.990 (0.970, 1.010)	0.307	0.667
22F	1.001 (0.982, 1.020)	0.926	0.926
23F	0.988 (0.969, 1.007)	0.208	0.667
33F	0.989 (0.970, 1.008)	0.271	0.667

Note: GMRs represent the adjusted geometric mean ratio in post-primary serotype-specific pneumococcal IgG concentration associated with each 2-fold increase in baseline anti-diphtheria toxoid IgG. Post-primary pneumococcal IgG, baseline anti-diphtheria toxoid IgG, and baseline serotype-specific pneumococcal IgG concentrations were log2-transformed before analysis. Models were adjusted for baseline serotype-specific pneumococcal IgG concentration and IPV treatment group. FDR, false discovery rate; GMR, geometric mean ratio; IgG, immunoglobulin G.

## Data Availability

The relevant data are presented in the manuscript. Additional datasets supporting the findings of this study may be made available to qualified researchers who submit a methodologically sound research proposal. Requests should be directed to the corresponding author. If access is approved, the data may be provided following appropriate de-identification and in accordance with applicable privacy laws, data-protection requirements, and participant consent.

## Supplementary Materials

The following supporting information can be downloaded at https://www.mdpi.com/article/10.3390/vaccines14100854/s1, File S1: List of investigators, study sites, and Ethics Committee (EC) approval dates; File S2: Eligibility criteria—inclusion and exclusion criteria; File S3: Composition of vaccines co-administered with 14-valent pneumococcal conjugate vaccine; Table S4: Post-vaccination serotype-specific pneumococcal IgG GMCs (µg/mL) and GMFRs by tertile-based baseline anti-diphtheria IgG groups; Table S5: Stratified sensitivity analysis evaluating the association between baseline anti-diphtheria toxoid IgG and post-primary pneumococcal IgG responses by baseline pneumococcal IgG status; File S6: ELISA method for measurement of anti-pneumococcal capsular polysaccharide IgG concentrations; Table S7: Post-primary pneumococcal IgG responses relative to the seroresponse threshold of 0.35 µg/mL by baseline anti-diphtheria IgG group; Figure S8: Pairwise correlations among post-primary serotype-specific pneumococcal IgG responses.
