# Supplementary material for: Association Between Pre-Existing Anti-Diphtheria Toxoid IgG and Immune Responses to a CRM197-Conjugated 14-Valent Pneumococcal Conjugate Vaccine in Infants: A Post Hoc Analysis of a Multicenter Phase III Trial
Source: Vaccines (Basel). 2026 Sep 28;14(10):854. doi: 10.3390/vaccines14100854 (PMC13709474; doi:10.3390/vaccines14100854)
Supplement: Supplementary file 1 [file vaccines-14-00854-s001.zip › vaccines-4573381-supplementary.pdf]

Supplementary Material

File S1: List of Investigators, Study sites, Ethics Committee (EC) approval dates

Investigator`s Name	Site Name	EC Registration Number	EC Approval Date
Dr. Nagarjuna Chadalavada	Aditya Multispecialty Hospital, Guntur	ECR/1347/Inst/AP/2020	24-03-2025
Dr. Atul Jindal	All India Institute of Medical Sciences (AIIMS), Raipur	ECR/714/Inst/CT/2015/RR-21	08-05-2025
Dr. Bhagirathi Dwibedi	All India Institute of Medical Sciences (AIIMS), Bhubaneswar	ECR/534/Inst/OD/2014/RR-20	04-07-2025
Dr. Shailesh Patil	Belagavi Institute of Medical Sciences, Belgaum	ECR/801/Inst/KA/2016/RR-20	15-04-2025
Dr. Ramesh.M	BGS Global Institute of Medical Sciences, Bangalore	ECR/1307/Inst/KA/2019	20-06-2025
Dr. Sonali Hemant Palkar	Bharati Vidyapeeth Medical College Hospital, Pune	ECR/313/Inst/MH/2013/RR-24	26-05-2025
Dr. Pradeep.N	Cheluvamba Hospital, Mysore	ECR/134/Inst/KA/2013/RR-19	27-06-2025
Dr. Jaishree Vasudevan	Chettinad Hospital & Research Institute, Chennai	ECR/1589/Inst/TN/2021	24-03-2025
Dr. Kishori Sharan Agarwal	Chirayu Hospital, Jaipur	ECR/1582/Inst/RJ/2021	19-03-2025
Dr. B. Vijai Anand Babu	Government General Hospital, Kurnool	ECR/1397/Inst/AP/2020	15-04-2025
Dr. Pareshkumar.A.Thakkar	Govt. Medical College & SSG General Hospital, Vadodara	ECR/85/Inst/GJ/2013/RR-24	21-04-2025
Dr. Jai Prakash Narayan	JLN Medical College, Ajmer	ECR/1156/Inst/RJ/2018/RR-22	15-03-2025
Dr. M.D. Ravi	JSS Hospital, Mysuru	ECR/387/Inst/KA/2013/RR-22	13-06-2025
Dr. B. S. Chakravarthy	King George Hospital, Visakhapatnam	ECR/197/Inst/KGH/2013/RR-20	27-05-2025

Investigator`s Name	Site Name	EC Registration Number	EC Approval Date
Dr. N. S. Mahantshetti	KLEs Dr. Prabhakar Kore Hospital, Belagavi	ECR/211/Inst/KA/2013/RR-24	21-07-2025
Dr. Mohit Poddar	Maharaja Agrasen Superspeciality Hospital, Jaipur	ECR/1222/Inst/RJ/2019/RR-22	18-03-2025
Dr. Jog Pramod Prabhakar	Medipoint Hospitals, Pune	ECR/357/Inst/MH/2013/RR-20	06-03-2025
Dr. Girish Nanoti	NKP Salve Institute of Medical sciences & Research Centre & Lata Mangeshkar Hospital, Nagpur	ECR/88/Inst/MH/2013/RR-24	25-06-2025
Dr. Paruchuri Anil Kumar	Old Government General Hospital (Associated By Govt. Siddhartha Medical College), Vijayawada	ECR/633/Inst/AP/2014/RR-19	26-03-2025
Dr. Ramanath Andy Karayalar	Panimalar Medical College Hospital & Research Institute, Chennai	ECR/1399/Inst/TN/2020	24-03-2025
Dr. Savita Verma	PT. B D Sharma Post Graduate Institute of Medical Sciences, Rohtak	ECR/293/Inst/HR/2013/RR-19	04-07-2025
Dr. Dhongade Ramchandra Keshav	Sant Dnyaneshwar Medical Education & Research Centre, Pune	ECR/127/Inst/MH/2013/RR-19	09-04-2025
Dr. Neeraj Kumar	Sarojini Naidu Medical College & Hospital, Uttar Pradesh	ECR/1409/Inst/UP/2020	28-04-2025
Dr. Madhukar Pandey	Shubham Sudbhawana Superspeciality Hospital, Varanasi	ECR/667/Inst/UP/2014/RR-20	22-03-2025
Dr. Patil Shyamkant Suresh	Signus Hospital, Nashik	ECR/1733/Inst/MH/2022	16-04-2025
Dr. Javadekar Bakul Balchandra	Smt. B. K. Shah Medical Institute & Research Centre, Vadodara	ECR/152/Inst/GJ/2013/RR-24	13-08-2025

Investigator`s Name	Site Name	EC Registration Number	EC Approval Date
Dr. Sarala Premkumar	Sri Ramachandra Hospital, Chennai	ECR/203/Inst/TN/2013/RR-24	13-06-2025
Dr. G. Balakishor	St. Theresa's Hospital (STH), Sanath Nagar	ECR/230/Inst/AP/2013/RR-22	25-03-2025
Dr. Manish Narang	UCMS & GTB Hospital, Delhi	ECR/510/Inst/DL/2014/RR-20	26-04-2025

EC: Ethics Committee

File S2: Eligibility criteria – Inclusion and Exclusion criteria

Inclusion Criteria:

Participants who had met all the following inclusion criteria were selected for study participation.:

1. Participants' parent(s)/LAR(s) who, in the opinion of the investigator, can and will comply, with the requirements of the protocol (e.g., completion of the diary cards, return for follow-up visits, with access to a consistent means of telephone contact, either residential landline or mobile).
2. Written or thumb printed informed consent (including audio-visual recording of consent process) obtained from the parent(s)/LAR(s) of the participant prior to performing any study specific procedure.
3. A male or female healthy infant of either gender between 6-8 weeks (42-56 days, both inclusive) of age at the time of first vaccination.
4. Healthy infants with body weight ≥ 3300 gms at the time of first vaccination.
5. Good clinical condition established by medical history and physical examination (with no acute disease, infection or high temperature) as judged by the principal investigator.
6. Participants not participating in any other clinical trials.
7. Infants with a minimal vaccination status for their age at the time of enrolment (Receipt of single birth dose of BCG, OPV and HBV vaccines (within 14 days of birth)).
8. Infants without contraindications or precautionary circumstances for participating in the trial.

Exclusion Criteria:

The following criteria were checked at the time of study entry. Participants who had met any of the following exclusion criteria were not enrolled in the study.:

1. Child in care, defined as a child who has been placed under the control or protection of an agency, organisation, institution or entity by the courts, the government or a government body, acting in accordance with powers conferred on them by law or regulation. The definition of a child in care can include a child cared for by foster parents or living in a care home or institution, provided that the arrangement falls within the definition above. The definition of a child in care does not include a child who is adopted or has an appointed legal guardian.
2. Prior immunization or intent to receive oral/injectable polio vaccine (OPV/IPV) within the study period with an exception of birth dose of oral polio vaccine.
3. Current illness (especially fever) or any acute or congenital illness or disability.
4. Use of any investigational or non-registered product (drug or vaccine) other than the study vaccine during the period starting 30 days before the administration of study vaccine (Day -29 to Day 0), or planned use during the study period.
5. Participants receiving immunosuppressive therapy
6. Any medical condition that in the judgment of the investigator would make intramuscular injection unsafe.
7. Known or suspected allergy to any of the vaccine components. History of allergic disease or history of a serious reaction to any prior vaccination or known hypersensitivity likely to be exacerbated by any component of the study vaccines.
8. Any sign or symptom or systemic dysfunction, especially of the central nervous system (CNS)
9. Known family history of SIDS (Sudden Infant Death Syndrome).
10. Planned or elective surgery during the course of the study.
11. Infants who have received any blood products, any dose of corticosteroids, cytotoxic agents or radiotherapy.
12. Participants and / or their mothers who have participated in another clinical trial of an investigational agent within last 30 days or likely to participate during the study course.
13. Inability or unwillingness of the parent to abide by the requirements of the protocol.

14. Any confirmed or suspected immunosuppressive or immunodeficient condition, based on medical history and physical examination (no laboratory testing required).
15. Family history of congenital or hereditary immunodeficiency.
16. Administration of immunoglobulins and/or any blood products during the period starting 30 days before the administration of study vaccine or planned administration during the study period.
17. Administration of long-acting immune-modifying drugs during the period starting 30 days prior to the proposed first vaccine dose or at any time during the study period.
18. Acute disease and/or fever at the time of vaccination.
    - Fever is defined as the endogenous elevation of at least one measured body temperature of ≥ 38°C (≥ 100.4°F).
19. Infant is a direct descendant (child or grand-child) of any person employed by the Sponsor, the Contract Research Organization (CRO) or the Study Site (including the PI and study site personnel).
20. Any criteria, which in the judgement of the Investigator, suggests that the participant would not be compliant with the study protocol.

File S3: Composition of vaccines co-administered with 14-valent pneumococcal conjugate vaccine

DTwP-HepB-Hib (ComBE Five®)

Each dose of 0.5 mL contains

Diphtheria Toxoid…………………………….....25 Lf (≥30 IU)

Tetanus Toxoid………………………………...5.5 Lf (≥60 IU)

B. Pertussis (Whole Cell) ……………………...16 IOU (≥4 IU)

r-HBsAg……………………………………...12.5 µg

Purified Capsular Polysaccharide

Of Hib (PRP) covalently linked

to 20 to 36.7 µg of Tetanus Toxoid………………11 µg

Al^{+++} ……………………………………………… ≤ 1.25 mg

Thiomersal………………………………………… 0.01% w/v

IPV

Bio E's Inactivated Poliomyelitis Vaccine, Adsorbed (Test Vaccine)

Each 0.5 mL dose of vaccine contains

Inactivated Poliomyelitis virus Type 1

(S19/MahP1/N18S)# ………………………....20 D-antigen units

Inactivated Poliomyelitis virus Type 2

(S19/MEF2P1/N18S)# ………………………..4 D-antigen units

Inactivated Poliomyelitis virus Type 3

(S19/SktP1/N18S)# …………………………....16 D-antigen units

Aluminium Hydroxide Gel

(equivalent to Al+++) …………………………..≤1.25 mg

2-Phenoxyethanol as preservative………..4 mg

Produced in Vero cells

Poliomyelitis vaccine bulks (Types 1, 2 and 3) were inactivated using formaldehyde

Sanofi's Inactivated Poliomyelitis Vaccine – IMOVAX Polio (Comparator)

Each 0.5 mL dose of vaccine contains

Poliomyelitis virus (inactivated)

Type 1 (Mahoney strain) #.......................40 DU*+

Type 2 (MEF-1 strain) # ....8 DU*+

Type 3 (Saukett strain) # ....32 DU*+

# produced on VERO cells

* DU: D-antigen unit

+ or equivalent antigenic quantity determined by a suitable immunochemical method

Rotavirus (ROTAVAC 5D®): Rotavirus Vaccine (Live Attenuated, Oral) IP

Each dose of 0.5 mL contains:

Vero cell derived Rotavirus 116E bulk, Live attenuated ………… NLT $10^{5.0}$ FFU

Neomycin Sulphate IP……………………………………………. 15 µg

Kanamycin Acid Sulphate IP …………………………………... 15 µg

Sucrose IP………………………………………………………… 0.25 gms

Trehalose BP…………………………………………………... 2.5 mg

Lactalbumin Hydrolysate (LAH) ………………………………… 2.5 mg

Human Albumin IP ……………………………………………….0.35%

Potassium Dihydrogen Orthophosphate BP ……………………... 1.65 mg

Dipotassium Hydrogen Orthophosphate BP …………………….. 10 mg

Trisodium Citrate Dihydrate IP …………………………………. 7.75 mg

Water for Injection IP …………………………………………… q.s.

Table S4: POST-VACCINATION SEROTYPE-SPECIFIC PNEUMOCOCCAL IgG GMCs (µg/mL) AND GMFRs BY TERTILE-BASED BASELINE ANTI-DIPHTHERIA IgG GROUPS

Serotype	Post-vac GMC in Low anti-D IgG (n=201)	Post-vac GMC in Medium anti-D IgG (n=201)	Post-vac GMC in High anti-D IgG (n=201)	p value (post-IgG)	GMFR Low anti-D IgG	GMFR Medium anti-D IgG	GMFR High anti-D IgG	p value (fold rise)
1	2.23	2.44	2.31	0.199	3.75	4.09	4.01	0.579
3	1.08	1.09	1.02	0.842	4.21	4.42	3.86	0.391
4	0.88	0.92	0.88	0.989	3.76	4.07	3.93	0.686
5	2.55	2.44	2.42	0.620	4.47	3.72	4.38	0.033
6B	3.01	3.08	3.10	0.787	3.79	3.71	3.83	0.649
7F	2.54	2.77	2.58	0.379	3.75	4.04	3.84	0.778
9V	1.75	1.67	1.59	0.678	4.07	3.64	3.99	0.551
14	6.58	7.02	6.81	0.425	3.71	3.56	3.50	0.884
18C	1.87	1.69	1.74	0.334	3.28	3.39	3.47	0.677
19A	3.51	3.68	3.32	0.351	3.68	3.57	3.71	0.936
19F	8.93	7.89	8.24	0.209	3.86	3.64	3.53	0.877
22F	4.65	4.47	4.47	0.807	3.78	3.39	4.05	0.110
23F	2.97	2.73	2.70	0.428	4.08	3.55	3.70	0.330
33F	2.41	2.46	2.28	0.761	3.86	4.00	3.92	0.990

Tertile cut-points for baseline anti-diphtheria toxoid IgG were ≤0.1219 IU/mL (low), >0.1219 to ≤0.6143 IU/mL (medium), and >0.6143 IU/mL (high). p values were obtained using the Kruskal-Wallis test applied to individual post-primary IgG concentrations and individual fold rises across the three tertile groups, respectively. GMC, geometric mean concentration; GMFR, geometric mean fold rise; IgG, immunoglobulin G.

Table S5: STRATIFIED SENSITIVITY ANALYSIS EVALUATING THE ASSOCIATION BETWEEN BASELINE ANTI-DIPHTHERIA TOXOID IGG AND POST-PRIMARY PNEUMOCOCCAL IGG RESPONSES BY BASELINE PNEUMOCOCCAL IGG STATUS

Serotype	Baseline pneumococcal IgG	N	Adjusted GMR (95% CI)	p value	FDR q value
1	<0.35	147	1.006 (0.952, 1.062)	0.839	0.923
1	≥0.35	456	1.017 (0.992, 1.042)	0.188	0.664
3	<0.35	349	0.969 (0.931, 1.008)	0.115	0.644
3	≥0.35	254	0.973 (0.942, 1.004)	0.089	0.644
4	<0.35	402	1.010 (0.983, 1.037)	0.460	0.805
4	≥0.35	201	0.992 (0.952, 1.033)	0.682	0.909
5	<0.35	146	1.027 (0.982, 1.075)	0.240	0.664
5	≥0.35	457	0.979 (0.957, 1.001)	0.062	0.644
6B	<0.35	90	1.015 (0.952, 1.083)	0.648	0.909
6B	≥0.35	513	0.997 (0.976, 1.018)	0.769	0.923
7F	<0.35	116	1.027 (0.975, 1.081)	0.311	0.664
7F	≥0.35	487	1.010 (0.988, 1.032)	0.395	0.738
9V	<0.35	206	0.994 (0.960, 1.029)	0.732	0.923
9V	≥0.35	397	0.980 (0.956, 1.004)	0.107	0.644
14	<0.35	19	0.959 (0.792, 1.162)	0.651	0.909
14	≥0.35	584	1.013 (0.992, 1.035)	0.221	0.664
18C	<0.35	166	0.996 (0.949, 1.044)	0.857	0.923

Serotype	Baseline pneumococcal IgG	N	Adjusted GMR (95% CI)	p value	FDR q value
18C	≥0.35	437	0.990 (0.971, 1.009)	0.309	0.664
19A	<0.35	74	0.997 (0.935, 1.062)	0.921	0.955
19A	≥0.35	529	0.987 (0.968, 1.007)	0.205	0.664
19F	<0.35	20	0.981 (0.789, 1.219)	0.852	0.923
19F	≥0.35	583	0.993 (0.972, 1.014)	0.507	0.836
22F	<0.35	49	0.951 (0.870, 1.040)	0.264	0.664
22F	≥0.35	554	1.000 (0.980, 1.020)	0.986	0.986
23F	<0.35	103	0.988 (0.934, 1.045)	0.664	0.909
23F	≥0.35	500	0.990 (0.969, 1.011)	0.332	0.664
33F	<0.35	134	1.030 (0.980, 1.083)	0.247	0.664
33F	≥0.35	469	0.980 (0.959, 1.002)	0.076	0.644

Baseline serotype-specific pneumococcal IgG concentration was used to define the strata (<0.35 or ≥0.35 µg/mL). Within each stratum, post-primary pneumococcal IgG and baseline anti-diphtheria toxoid IgG concentrations were log2-transformed. Adjusted GMRs represent the relative change in post-primary serotype-specific pneumococcal IgG concentration associated with each 2-fold increase in baseline anti-diphtheria toxoid IgG and were obtained from linear regression models adjusted for IPV treatment group. p values correspond to the baseline anti-diphtheria toxoid IgG coefficient in these models. FDR q values were calculated using the Benjamini-Hochberg procedure across the 28 stratum-specific serotype analyses. CI, confidence interval; FDR, false discovery rate; GMR, geometric mean ratio; IgG, immunoglobulin G.

File S6: ELISA method for measurement of anti-Pneumococcal Capsular Polysaccharide IgG concentrations

Serotype-specific anti-pneumococcal capsular polysaccharide IgG concentrations were measured using an enzyme-linked immunosorbent assay developed at CRL, KIMS, Bengaluru, based on the WHO training manual for pneumococcal ELISA, with minor modifications. No commercial ELISA kit was used. The assay was validated for accuracy, precision, range, linearity, stability, and robustness. The 1st International Standard for Human Anti-pneumococcal Capsular Reference Serum, 007sp, supplied by the National Institute for Biological Standards and Control, United Kingdom, was used as the reference standard, and serotype-specific antibody concentrations were determined relative to its assigned IgG concentrations. Dilution-corrected antibody concentrations were calculated using a 4-parameter logistic regression model.

Briefly, pre-vaccination and post-vaccination human serum samples, reference serum, and quality-control serum were pre-absorbed with CWPS and PnPs 22F for all the serotypes except 22F. For serotype 22F, human serum samples, reference serum and quality control serum were

preabsorbed with CWPS Multi™ to remove cell-wall polysaccharide-reactive antibodies. The pre-absorbed sera were then added to ELISA plates coated with pneumococcal capsular polysaccharide of the respective serotype. Bound serotype-specific anti-pneumococcal IgG antibodies were detected using goat anti-human IgG alkaline phosphatase conjugate. Following addition of pNPP chromogenic substrate, optical density was measured at 405 nm with 690 nm as the reference wavelength. Corrected optical density was calculated as OD405 − OD690 and was used to determine serotype-specific IgG concentrations from the reference curve.

Table S7. TABLE: POST-PRIMARY PNEUMOCOCCAL IGG RESPONSES RELATIVE TO THE SERORESPONSE THRESHOLD OF 0.35 µg/mL BY BASELINE ANTI-DIPHTHERIA IgG GROUP

Serotype	Low anti-D IgG <0.1 IU/mL: <0.35 µg/mL n/N (%)	Low anti-D IgG: ≥0.35 µg/mL n/N (%)	Medium anti-D IgG 0.1 to <1.0 IU/mL: <0.35 µg/mL n/N (%)	Medium anti-D IgG: ≥0.35 µg/mL n/N (%)	High anti-D IgG ≥1.0 IU/mL: <0.35 µg/mL n/N (%)	High anti-D IgG: ≥0.35 µg/mL n/N (%)
1	1/191 (0.5%)	190/191 (99.5%)	5/307 (1.6%)	302/307 (98.4%)	1/105 (1.0%)	104/105 (99.0%)
3	22/191 (11.5%)	169/191 (88.5%)	37/307 (12.1%)	270/307 (87.9%)	15/105 (14.3%)	90/105 (85.7%)
4	13/191 (6.8%)	178/191 (93.2%)	18/307 (5.9%)	289/307 (94.1%)	7/105 (6.7%)	98/105 (93.3%)
5	1/191 (0.5%)	190/191 (99.5%)	0/307 (0.0%)	307/307 (100.0%)	1/105 (1.0%)	104/105 (99.0%)
6B	1/191 (0.5%)	190/191 (99.5%)	1/307 (0.3%)	306/307 (99.7%)	1/105 (1.0%)	104/105 (99.0%)
7F	0/191 (0.0%)	191/191 (100.0%)	0/307 (0.0%)	307/307 (100.0%)	0/105 (0.0%)	105/105 (100.0%)
9V	1/191 (0.5%)	190/191 (99.5%)	3/307 (1.0%)	304/307 (99.0%)	3/105 (2.9%)	102/105 (97.1%)
14	0/191 (0.0%)	191/191 (100.0%)	0/307 (0.0%)	307/307 (100.0%)	0/105 (0.0%)	105/105 (100.0%)
18C	1/191 (0.5%)	190/191 (99.5%)	5/307 (1.6%)	302/307 (98.4%)	0/105 (0.0%)	105/105 (100.0%)
19A	2/191 (1.0%)	189/191 (99.0%)	1/307 (0.3%)	306/307 (99.7%)	0/105 (0.0%)	105/105 (100.0%)
19F	0/191 (0.0%)	191/191 (100.0%)	0/307 (0.0%)	307/307 (100.0%)	0/105 (0.0%)	105/105 (100.0%)
22F	0/191 (0.0%)	191/191 (100.0%)	1/307 (0.3%)	306/307 (99.7%)	0/105 (0.0%)	105/105 (100.0%)
23F	0/191 (0.0%)	191/191 (100.0%)	3/307 (1.0%)	304/307 (99.0%)	0/105 (0.0%)	105/105 (100.0%)
33F	1/191 (0.5%)	190/191 (99.5%)	3/307 (1.0%)	304/307 (99.0%)	0/105 (0.0%)	105/105 (100.0%)

Values are n/N (%). Post-primary samples were collected 28 days after dose 3. Baseline anti-diphtheria IgG groups: low, <0.1 IU/mL; medium, 0.1 to <1.0 IU/mL; high, ≥1.0 IU/mL.

Figure S8. Figure. PAIRWISE CORRELATIONS AMONG POST-PRIMARY SEROTYPE-SPECIFIC PNEUMOCOCCAL IgG RESPONSES.

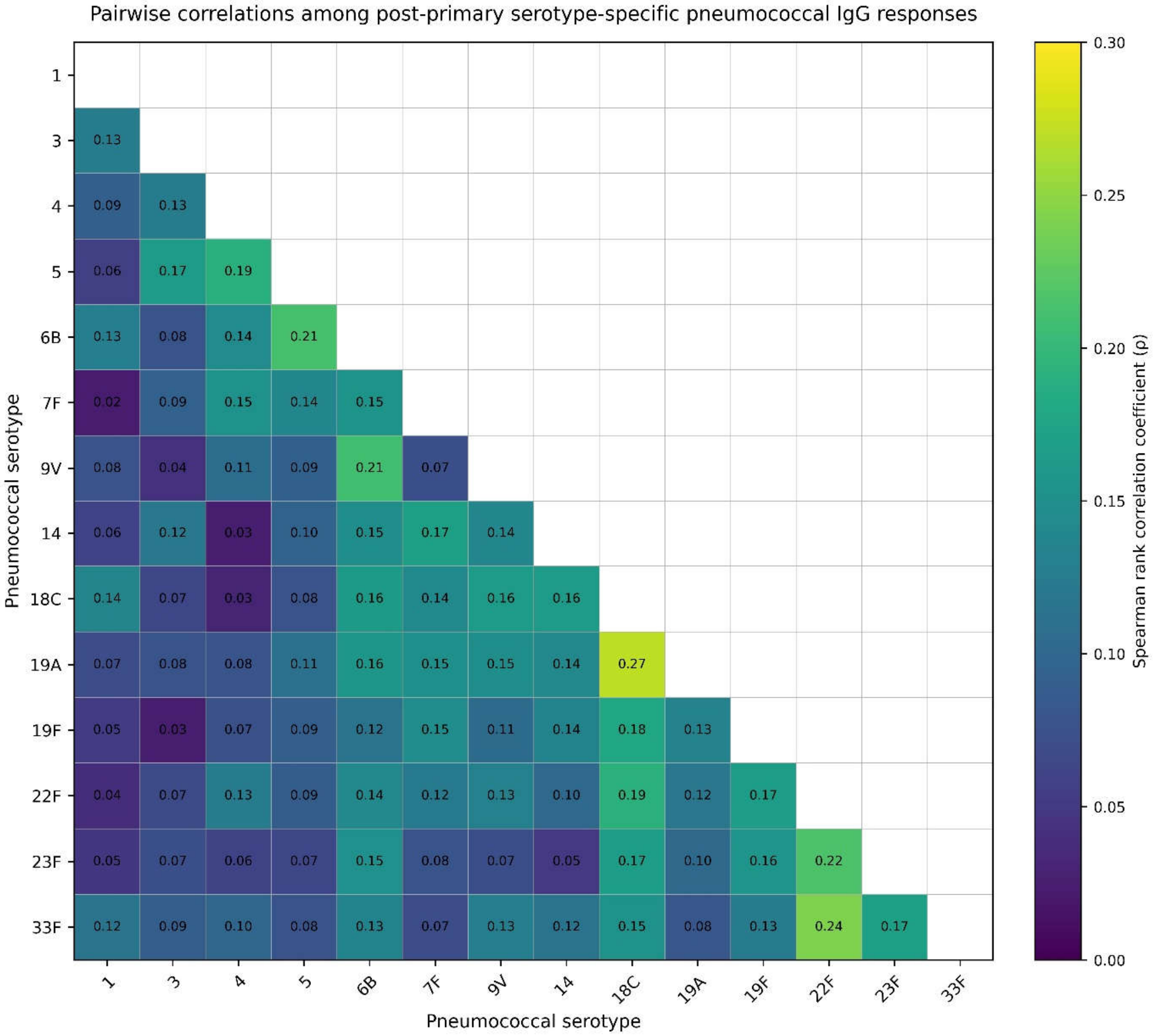

	1	3	4	5	6B	7F	9V	14	18C	19A	19F	22F	23F	33F
1														
3	0.13													
4	0.09	0.13												
5	0.06	0.17	0.19											
6B	0.13	0.08	0.14	0.21										
7F	0.02	0.09	0.15	0.14	0.15									
9V	0.08	0.04	0.11	0.09	0.21	0.07								
14	0.06	0.12	0.03	0.10	0.15	0.17	0.14							
18C	0.14	0.07	0.03	0.08	0.16	0.14	0.16	0.16						
19A	0.07	0.08	0.08	0.11	0.16	0.15	0.15	0.14	0.27					
19F	0.05	0.03	0.07	0.09	0.12	0.15	0.11	0.14	0.18	0.13				
22F	0.04	0.07	0.13	0.09	0.14	0.12	0.13	0.10	0.19	0.12	0.17			
23F	0.05	0.07	0.06	0.07	0.15	0.08	0.07	0.05	0.17	0.10	0.16	0.22		
33F	0.12	0.09	0.10	0.08	0.13	0.07	0.13	0.12	0.15	0.08	0.13	0.24	0.17	

Values shown are Spearman rank correlation coefficients (ρ) for each unique pair of BE-PCV14 serotypes. Correlation coefficients ranged from 0.024 to 0.271. The lower triangle is displayed; the upper triangle is omitted because it contains duplicate correlations.
